# SingmiR: a single-cell miRNA alignment and analysis tool

**DOI:** 10.1093/nar/gkae225

**Published:** 2024-04-04

**Authors:** Annika Engel, Shusruto Rishik, Pascal Hirsch, Verena Keller, Tobias Fehlmann, Fabian Kern, Andreas Keller

**Affiliations:** Chair for Clinical Bioinformatics, Saarland University, 66123 Saarbrücken, Germany; Chair for Clinical Bioinformatics, Saarland University, 66123 Saarbrücken, Germany; Chair for Clinical Bioinformatics, Saarland University, 66123 Saarbrücken, Germany; Chair for Clinical Bioinformatics, Saarland University, 66123 Saarbrücken, Germany; Chair for Clinical Bioinformatics, Saarland University, 66123 Saarbrücken, Germany; Chair for Clinical Bioinformatics, Saarland University, 66123 Saarbrücken, Germany; Department of Clinical Bioinformatics (CLIB), Helmholtz Institute for Pharmaceutical Research Saarland (HIPS), Helmholtz Centre for Infection Research, 66123 Saarbrücken, Germany; Chair for Clinical Bioinformatics, Saarland University, 66123 Saarbrücken, Germany; Department of Clinical Bioinformatics (CLIB), Helmholtz Institute for Pharmaceutical Research Saarland (HIPS), Helmholtz Centre for Infection Research, 66123 Saarbrücken, Germany

## Abstract

Single-cell RNA sequencing (RNA-seq) has revolutionized our understanding of cell biology, developmental and pathophysiological molecular processes, paving the way toward novel diagnostic and therapeutic approaches. However, most of the gene regulatory processes on the single-cell level are still unknown, including post-transcriptional control conferred by microRNAs (miRNAs). Like the established single-cell gene expression analysis, advanced computational expertise is required to comprehensively process newly emerging single-cell miRNA-seq datasets. A web server providing a workflow tailored for single-cell miRNA-seq data with a self-explanatory interface is currently not available. Here, we present SingmiR, enabling the rapid (pre-)processing and quantification of human miRNAs from noncoding single-cell samples. It performs read trimming for different library preparation protocols, generates automated quality control reports and provides feature-normalized count files. Numerous standard and advanced analyses such as dimension reduction, clustered feature heatmaps, sample correlation heatmaps and differential expression statistics are implemented. We aim to speed up the prototyping pipeline for biologists developing single-cell miRNA-seq protocols on small to medium-sized datasets. SingmiR is freely available to all users without the need for a login at https://www.ccb.uni-saarland.de/singmir.

## Graphical Abstract

**Graphical Abstract:**
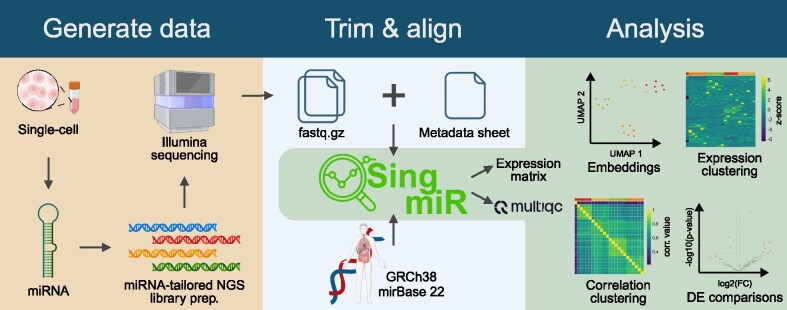

## Introduction

One of the best studied classes of noncoding RNAs is microRNAs (miRNAs), 20–25 nt long molecules that regulate potentially up to 60% of the coding genes found in humans, either degrading messenger RNA (mRNA) or repressing protein translation, mainly through 3′-UTR (untranslated region) binding (1,2). miRNAs play a key role in the regulation of cell states and are increasingly relevant biomarkers in new disease diagnostic and therapeutic approaches, e.g. the overexpression of miRNAs in lymphomas (3) or in cancer (4). Despite their importance and possible improvement to diagnostics, we are currently limited to quantifying miRNAs with bulk sequencing experiments only. For instance, we know the mRNA expression patterns of circulating tumor cells from single-cell studies (5) but so far could not study in detail, through bulk sequencing alone, how the miRNome is shaping rare cell populations. Besides, miRNAs regulate genes and pathways (6), the analysis of which is typically accomplished with software tools (7,8) that are well established for bulk studies but so far are not tailored for single-cell sequencing data. To date, depth and availability of single-cell miRNA sequencing (miRNA-seq) datasets are lacking behind their mRNA counterparts, primarily due to persisting experimental challenges, which render the selective enrichment and subsequent sequencing of miRNA molecules an intricate affair. However, a few protocols exist that are continuously optimized for a better quantitative yield, while there is no commercial option currently on the market. Attempts to estimate miRNA abundance in isolated cells based on primary miRNA expression in single-nucleus sequencing data have produced extremely sparse count matrices that show high variability (9). Even at the higher RNA quantities of standard bulk sequencing approaches, it can be challenging to quantify miRNA levels accurately. For example, the presence of other classes of noncoding RNA (10–12) and the formation of adapter dimers (13) are inevitable sources of bias. Existing technical issues are further complicated by the small RNA input quantities typically required for high-resolution single-cell libraries. The primary ideas to tackle these challenges are to remove excess adapters by combining digestion and size selection (14,15), reduction of adapter dimer formation by adapter chemical modification (16), mitigation of ligation bias by introducing degenerated bases to adapters (11) and polyadenylation (17). Performance optimization is therefore an ongoing effort, for which a recent benchmark provided new quantitative and qualitative grounds (18).

There exists a whole ecosystem of tools to analyze miRNA data: stand-alone tools such as miRDeep* (19) and web services such as miRMaster2.0 (20), sRNAbench/sRNAtoolbox (21), CBS-miRSeq (22) and others [a complete list is provided in miRMaster (20)]. However, currently no such tools exist for single-cell data analysis, which is projected to rapidly increase in the next few years due to newly developed protocols (18). The above-mentioned standard tools cannot be used right away for the single-cell analysis because they do not support the specific parameters used in such protocols, especially regarding the different adapter and barcode sequences as well as unique molecular identifier (UMI) layouts required.

We thus made our analysis pipeline available through a web server. We include the option to perform common comparative analyses, for instance embeddings by popular dimension reduction techniques, correlation and expression-based clustering, differential expression (DE) analyses and more. We aim to enable life science researchers planning to analyze and compare different single-cell miRNA-seq datasets with the necessary toolset, without requiring any computational or bioinformatics expertise.

## Materials and methods

The computational workflow of SingmiR consists of two main stages. First, the alignment and trimming pipeline, which removes the adapters specific to the library preparation method used, aligns the reads to the human genome and quantifies miRNA abundances. Second, an optional analysis pipeline computes overview plots and statistics for the processed user dataset. A comprehensive submission interface guides the user through the necessary steps, such as providing data and specifying details for an optional in-depth analysis. The results page allows to download all results for both computational pipelines and displays multiple adjustable visualizations.

### Alignment pipeline

SingmiR accepts inputs in the form of gzip compressed fastq files with the option to include a metadata file for downstream analysis. In the current implementation, each fastq file corresponds to one biological cell. Once uploaded, the data are extracted and the UMI sequence is added to the fastq file headers using a Python (version 3.12) script. It is trimmed using cutadapt, version 2.10 (23), which utilizes the Illumina Universal adapter sequence, pcr primer sequence and truseq adapter sequences along with the adapter parameters uploaded with the fastq files. The details of the trimming are stated in Table 1. Fastqc, version 0.11.8 (https://www.bioinformatics.babraham.ac.uk/projects/fastqc/) is used to perform quality metric checking for trimmed and raw reads. For the miRNA quantification, the cutadapt cleaned reads are mapped using bowtie, version 1.3.1 (24) against the human-derived miRNA from miRBase V22 (25) with 15-bp flanks. Fumi_tools, version 0.12.2 (https://ccb-gitlab.cs.uni-saarland.de/tobias/fumi_tools) is used to deduplicate the resulting bam files and in-house scripts were used to produce count and normalized matrices. Due to the recent emergence of transfer RNA-derived fragments (tRF) as a noncoding RNA regulator and their structural similarity to miRNAs (26), tRFs were detected by mapping against transfer RNA with bowtie, subsequent deduplication with Fumi_tools and finally quantified using MINTmap 1.0 (27). The quantification for tRFs is made separately available as downloadable count matrix.

**Table 1.** Trimming parameters and metadata from the case study required for SingmiR

Protocol	3′ Adapter	5′ Adapter	Method	UMI length
SB	Sandberg	Sandberg	SB	8
SB_4N	4N	4N	SB	8
SB_CL	CleanTag	CleanTag	SB	8
SB_C3	Sandberg	C3	SB	6
SBN	Sandberg	Sandberg	SBN	8
SBN_4N	4N	4N	SBN	8
SBN_CL	CleanTag	CleanTag	SBN	8
CL	CleanTag	CleanTag	CleanTag	8
CL_16C	CleanTag	CleanTag	CleanTag	8
CL_4N	4N	4N	CleanTag	8
CL_Block	CleanTag	Block	CleanTag	6
CL_C3	CleanTag	C3	CleanTag	6
CL_Rand	Rand	CleanTag	CleanTag	6
CL_SB	Sandberg	Sandberg	CleanTag	8
CL_UMI6	CleanTag	CleanTag UMI6	CleanTag	8
4N	4N	4N	4N	0
4N_C3	4N	C3	4N	0
4N_CL	CleanTag	CleanTag	4N	0
CATS	CATS	CATS	CATS	0

To quickly quantify all other classes of RNA, the cutadapt cleaned reads are also mapped using the STAR algorithm, version 2.5.3a (28) against an index of the human genome (GRCh38) using the parameters --outSAMstrandField intronMotif --outFilterMultimapNmax 50 --outFilterScoreMinOverLread --outFilterMultimapScoreRange 0 --outFilterMatchNmin 18 --outFilterMatchNminOverLread 0 --outFilterMismatchNoverLmax 0.04 --alignIntronMax 1. Next, the generated bam file is compared against the GRCh38 annotation in order quantify the reads mapped to any gene in the human genome. The deduplicated and mapped reads are compared against annotations extracted from GENCODE v25, piRBase v1 and GtRNAdb v18.1, overall containing rRNA, Mt rRNA, snoRNA, snRNA, sRNA, scaRNA, scRNA, piRBase, misc RNA, ribozyme, coding exons, lncRNA, ncRNA and protein-coding genes, using the featureCounts [included in subread in version 1.5.2 (29)] using the options -F SAF -O -M -f --fracOverlap 0 -s 0. We perform a reads per million mapped miRNA normalization to account for the differences in reads per file and coverage per miRNA. The trimming, mapping and feature count statistics are compiled into a MultiQC summary report in version 1.20.0 (30) and shared with the user. The rpmmm-normalized matrix is used to produce further downstream analyses according to the user selection. Both the MultiQC and the expression matrix files (miRNAs, other RNAs and tRFs) are available for download.

### Analysis pipeline

In addition to the raw data, a metadata sheet must be uploaded containing important sample parameters (one sample per row) and additional but optional descriptive information (column variables) for each sample. The user can select categories from the metadata sheet for an optional analysis of the miRNA features, as outlined in the following. To gain a deeper understanding of the dataset, we employ principal component analysis (PCA) and uniform manifold approximation and projection (UMAP) (31) as dimension reduction techniques. The resulting scatter plots are colored according to selected categories. In addition, a UMAP analysis is available for various preselected parameters to reveal higher order relationships between the samples and cells. We aim to discover batch effects and biologically relevant parameters by performing a principal variance component analysis (PVCA) on the selected categories. The residual category sums the variance in the data that cannot be associated with any of the categories provided in the metadata sheet.

A hierarchical clustering using the Euclidean distance and a complete linkage is performed on the standardized rpmmm-normalized log_2_-transformed expression values and on the sample correlation values calculated according to either Pearson or Spearman. When using the expression values, we performed clustering for different feature sets, all miRNAs, only expressed miRNA and top miRNAs determined by the highest coefficient of variation. We also provide the corresponding *P*-values calculated with the R function cor.test and adjusted with the Benjamini–Hochberg procedure, which controls the false discovery rate at an alpha level, together with a correlation plot indicating the significance of each value (***P < 0.001, **P < 0.01 and *P < 0.05). All results are presented in the form of a heatmap.

Performing DE analysis for all possible categories comes at increased computational costs. Therefore, DE analysis is performed in an interactive manner where the user selects a comparison, and the results are calculated on demand. This way, any comparison deemed valuable can be explored later in more detail. Besides the fold changes, we provide the *P*-values and adjusted *P*-values for *t*-tests and Wilcoxon signed-rank tests. The user can choose between the Benjamini–Hochberg procedure and the Bonferroni correction at a default alpha level of 0.05, the latter of which is known for its strong regulation of family-wise error levels. Additional measures include the effect size according to Cohen’s *d* and the area under the receiver operator curve. Graphical representations in the form of volcano and scatter plots accompany the DE analysis in table form.

### Web server implementation

The web server providing the front end and the underlying mechanics utilizes the Django Python web framework, version 2.1.7 (https://djangoproject.com/) inside Docker containers (https://www.docker.com/). Following data submission, we use the task queue manager Celery, version 5.2.7 (http://docs.celeryproject.org) together with the in-memory data structure store Redis, version 5.0 (https://redis.io/) to efficiently handle concurrent tasks. Both the alignment and analysis employ a Snakemake pipeline, version 7.30.1 (32). Additionally, the front end of the website uses Bootstrap, version 5.1.3 (https://getbootstrap.com/) and Font Awesome, version 6.1.1 (https://fontawesome.com/) for design purposes, as well as jQuery, version 3.7.1 (https://jquery.com/).

## Results

**Figure 1.**
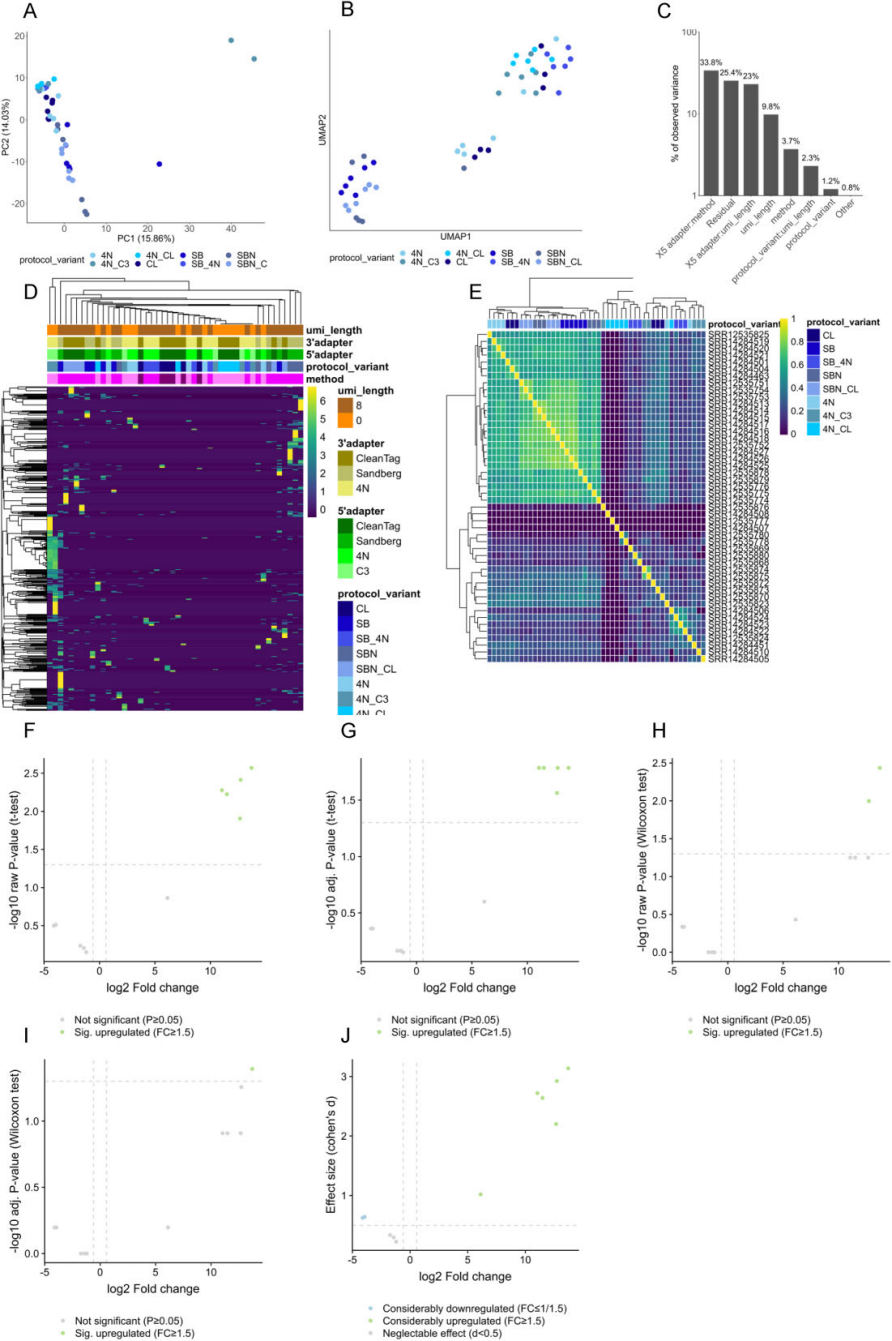
SingmiR results of the stage 2 dataset from (18) using the downstream analysis module. The comparison selected for the DE analysis is protocol ‘SBN CL’ versus ‘4N CL’. (**A**) Results of PCA. (**B**) Results of a UMAP analysis. (**C**) Results of the PVCA. (**D**) Results of the hierarchical clustering of the top 250 standardized log_2_-transformed rpmmm-normalized miRNA values selected by the coefficient of variation. (**E**) Sample correlation calculated with Pearson and grouped by hierarchical clustering. (**F**) Volcano plot of the raw *P*-values from a *t*-test. (**G**) Volcano plot of the adjusted *P*-values from a *t*-test (used adjusting method is the Benjamini–Hochberg procedure). (**H**) Volcano plot of the raw *P*-values from a Wilcoxon rank-sum test. (**I**) Volcano plot of the adjusted *P*-values from a Wilcoxon rank-sum test (used adjusting method is the Benjamini–Hochberg procedure). (**J**) Scatter plot showing the effect size (Cohen’s *d*) and the log_2_-transformed fold change.

To test the capabilities of our web server, raw data from a previously published high-quality single-cell miRNA-seq dataset were re-analyzed with SingmiR (18). This study covers different sample types, of which we first process the samples from the second stage. In detail, these are 48 samples equal to 48 single-cell profiles obtained from the human breast cancer cell line MCF7, generated with eight different protocols (six samples each). To visualize expression profiles, we consider the results of the downstream analysis module. In addition to this, users have several quality measurements available through the MultiQC report. We restrict our re-analysis here to a single category also examined by the original study, i.e. comparing different versions of the experimental protocol. PCA (Figure 1A) and UMAP (Figure 1B) provide an initial overview of the sample variability and clustering. Color legends of UMAP plots can be modified by various preselected parameters; to exemplify this, we show a single parameter specification. We recognize a clustering for some of the protocols, for example for the protocol ‘SBN_CL’. The PVCA indicates that the largest variance in the data can be found for the combination of the 5′ adapter and the method (33.8%) variables, next to the combination of the 5′ adapter with the UMI length (23%) (Figure 1C). A clustered heatmap of the log_2_-transformed expression reveals miRNA clusters across the samples/cells. Separately clustered heatmaps for different feature sets, i.e. using all given miRNAs, only the expressed miRNAs or the top miRNAs based on the highest variance across all cells, respectively, are computed automatically. Annotation bars above the plot highlight the sample/cell clustering of the individual categories for the top 250 miRNAs (see the ‘Materials and methods’ section) (Figure 1D). To investigate cell similarity, we calculate the Pearson correlation values for all miRNAs between all cell combinations and display a row- and column-clustered heatmap (Figure 1E). We observe a strong correlation between samples of the protocols ‘4N’, ‘SBN_CL’, ‘SBN’ and ‘SB’. DE analysis can be performed on demand for any category of interest with at least two groups. We compare ‘SBN_CL’ against ‘4N_CL’ using volcano plots for fold change against raw and adjusted *P*-values of a *t*-test (Figure 1F and G) and a Wilcoxon rank-sum test (Figure 1H and I). In addition, the effect size, which is calculated using Cohen’s *d*, is plotted against the fold change (Figure 1J) and indicates a considerable upregulation of seven miRNAs for the protocol ‘SBN_CL’. Yet only one significantly deregulated miRNA (hsa-miR-21-5p) remains for the adjusted *P*-value from the Wilcoxon rank-sum test and five significant deregulated miRNAs (hsa-miR-182-5p, hsa-miR-25-3p, hsa-miR-92a-3p, hsa-miR-183-5p and hsa-miR-21-5p) in the case of the *t*-test.

**Figure 2.**
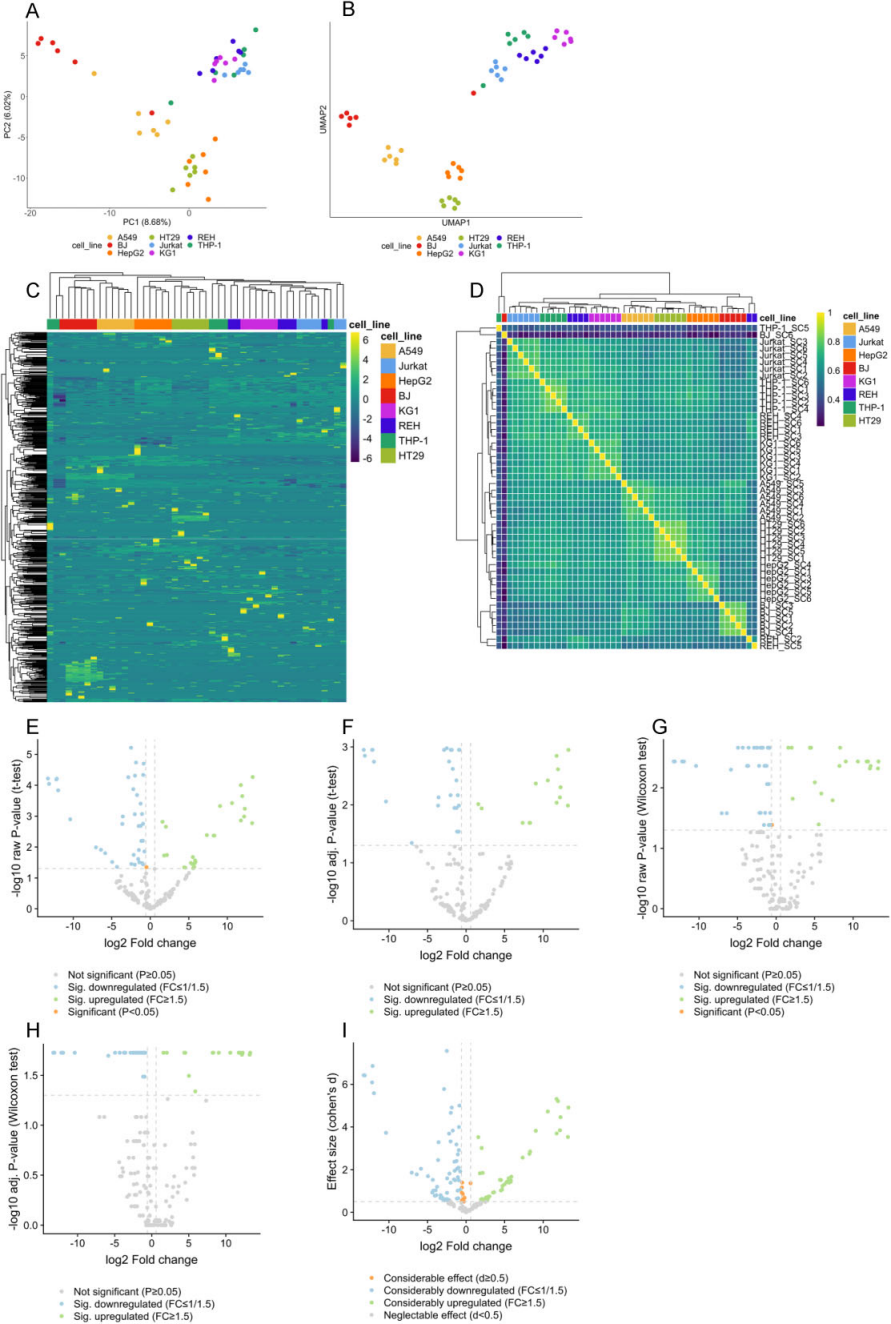
SingmiR results of the first stage 3 dataset from (18) using the downstream analysis module. The comparison selected for the DE analysis is cell line ‘HT29’ versus ‘KG1’. (**A**) Results of PCA. (**B**) Results of a UMAP analysis. (**C**) Results of the hierarchical clustering of the expressed standardized log_2_-transformed miRNAs selected by the coefficient of variation. (**D**) Sample correlation calculated with Pearson and grouped by hierarchical clustering. (**E**) Volcano plot of the raw *P*-values from a *t*-test. (**F**) Volcano plot of the adjusted *P*-values from a *t*-test (used adjusting method is the Benjamini–Hochberg procedure). (**G**) Volcano plot of the raw *P*-values from a Wilcoxon rank-sum test. (**H**) Volcano plot of the adjusted *P*-values from a Wilcoxon rank-sum test (used adjusting method is the Benjamini–Hochberg procedure). (**I**) Scatter plot showing the effect size (Cohen’s *d*) and the log_2_-transformed fold change.

In the study by Hücker *et al.*, another high-quality dataset is presented, consisting of 48 samples from 8 different cell lines (6 samples each), which was obtained using the most promising protocol ‘SBN_CL’. Thus, we also use this dataset to test our web server. The resulting embedding for PCA and UMAP shows a good separation of the samples by cell line origin (Figure 2A and B). This is also reflected in the clustering of the expression values and the correlation values (Figure 2C and D). As for this dataset there is only one categorical metadata variable with more than one level available, no PVCA can be performed. A DE analysis between the two cell lines ‘HT29’ and ‘KG1’ shows strong and significant fold changes, thus yielding numerous significantly deregulated miRNAs (Figure 2E–I). The results table for the comparison of the cell line ‘HT29’ to all other cell lines (‘A549’, ‘BJ’, ‘HepG2’, ‘Jurkat’, ‘KG1’, ‘REH’ and ‘THP-1’) is included in the supplementary material (Supplementary Table S1). By filtering for features that are significantly deregulated across all comparisons, we obtain three upregulated miRNAs (hsa-miRNA-200b-3p, hsa-miRNA-10a-5p and hsa-miRNA-141-3p). Remarkably, these miRNAs have been previously associated with human disease, namely in the context of colorectal and ovarian cancers (33–36). Consequently, it is plausible to observe a high expression predominantly in ‘HT29’, which had been derived from a human colon adenocarcinoma (37–40). While not pursued further in this work, the presented downstream analysis can serve as a starting point for further investigations.

## Discussion

The current absence of a graphical user interface (GUI) based single-cell RNA-seq analysis pipeline for noncoding RNAs so far required researchers to have sufficient computational expertise to eventually generate reliable results. As a comprehensive best-practice and easy-to-use workflow, the here presented web server will hopefully enhance comparability between novel datasets and facilitate fast pilot studies where the analysis is currently conducted by the experimenter (41,42). After having demonstrated the capabilities of SingmiR with previously published datasets (18), the here presented web server is well positioned to serve as a useful tool for upcoming and larger single-cell miRNA-seq studies. Additionally, the development of techniques that aim to predict miRNA activity based on single-cell mRNA data (43) indicates interest in this field. Recent advancements in sequencing technology (44) and the need to study miRNA activity in complex tissues contribute to the increasing emergence of single-cell studies (45). Therefore, we anticipate that our server will not only simplify the analysis of initial pilot stage projects but also serve as a stepping stone to a greater understanding of the single-cell miRNA landscape. Therefore, we hope to provide a useful tool to enable benchmarking studies for single-cell miRNA-seq, as previously done for small RNA-seq methods (11). While we currently only support low-throughput sequencing data, generating up to a few hundred thousand reads for one cell replicate per fastq file, the expansion to high-throughput data marks a promising way forward, supporting novel high-throughput protocols once they have been established in the single-cell community. To further advance the development of SingmiR, we encourage the community to provide us feedback and to propose new features of interest.

## Supplementary Material

gkae225_Supplemental_Files

## Data Availability

Data for the case study were made available by Hücker *et al.* on European Nucleotide Archive (https://www.ebi.ac.uk/ena/browser/home) under the project PRJNA659784.
