# Supplementary material for: SingmiR: a single-cell miRNA alignment and analysis tool
Source: Nucleic Acids Res. 2024 Apr 4;52(W1):W374–80. doi: 10.1093/nar/gkae225 (PMC11223861; doi:10.1093/nar/gkae225)
Supplement: gkae225_Supplemental_Files [file gkae225_supplemental_files.zip › Supplementary Table 1 legends.docx]

**Supplementary Table 1:** DE analysis result table from SingmiR of the first stage 3 dataset from (18) for the comparison of the cell line “HT29” with every other (“A549”, “BJ”, “HepG2”, “Jurkat”, “KG1”, “REH” and “THP-1”).
